# Supplementary material for: Targeted Polymer-Based Probes for Fluorescence Guided Visualization and Potential Surgery of EGFR-Positive Head-and-Neck Tumors
Source: Pharmaceutics. 2020 Jan 1;12(1):31. doi: 10.3390/pharmaceutics12010031 (PMC7022460; doi:10.3390/pharmaceutics12010031)
Supplement: Supplementary file 1 [file pharmaceutics-12-00031-s001.pdf]

pharmaceutics

Supplementary Materials: Targeted Polymer-Based Probes for Fluorescence Guided Visualization and Potential Surgery of EGFR-Positive Head-and-Neck Tumors

Robert Pola, Eliška Böhmová, Marcela Filipová, Michal Pechar, Jan Pankrác, David Větvička, Tomáš Olejár, Martina Kabešová, Pavla Poučková, Luděk Šefc, Michal Zábrodský, Olga Janoušková, Jan Bouček and Tomáš Etrych

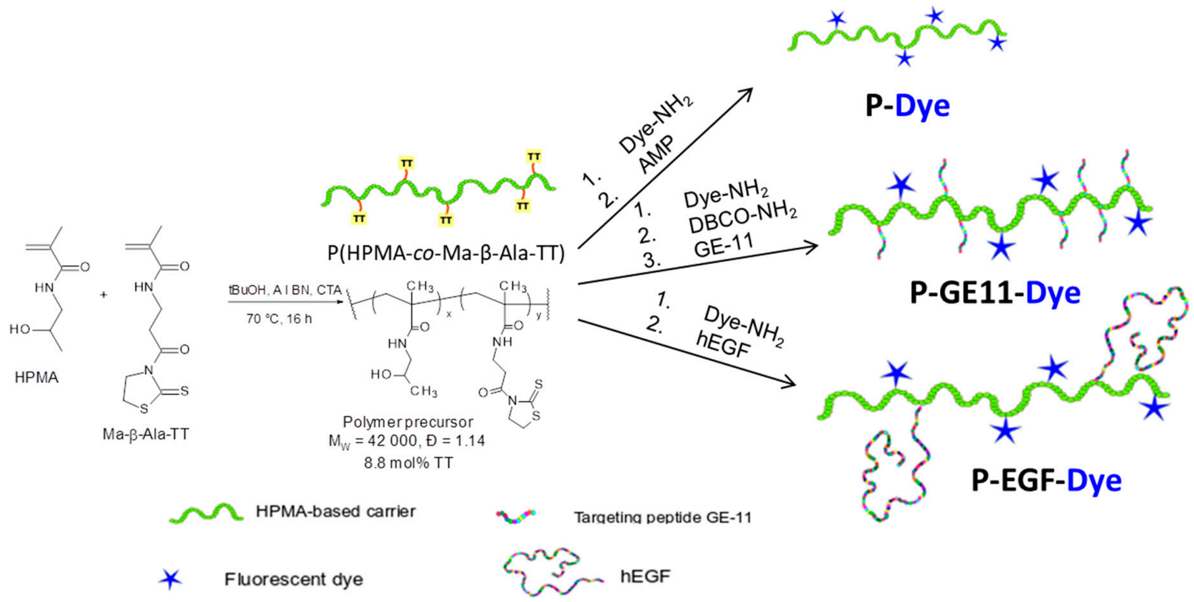

Figure S1. Schematic synthetic overview peptide-targeted polymer probes.

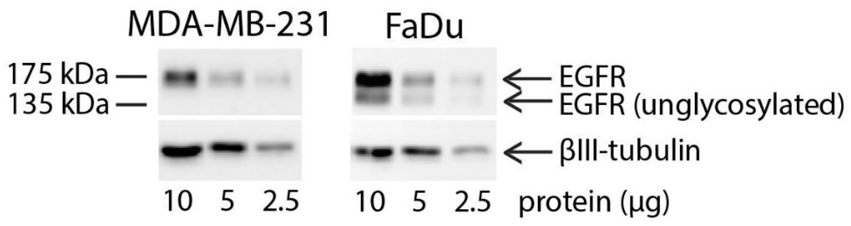

Figure S2. Western blot analysis of EGFR expression on FaDu a MDA cells.
